# Comparison of CEAC, BEAM and IEAC conditioning regimens followed by autologous stem cell transplantation in peripheral T-cell lymphoma patients

**DOI:** 10.1038/s41598-022-18540-x

**Published:** 2022-08-23

**Authors:** Yi-Ying Xiong, Jing Wang, Li Wang, Jian-Bin Chen, Lin Liu, Xiao-Qiong Tang, Xin Wang, Hong-Bin Zhang

**Affiliations:** Department of Hematology, The First Affiliated Hospital of Chongqing Medical University, Chongqing, 400016 China

**Keywords:** Stem cells, Risk factors, Cancer therapy

## Abstract

Autologous stem cell transplantation (ASCT) is an important treatment for peripheral T-cell lymphoma (PTCL) patients both during front and salvage therapy. In order to explore the appropriate conditioning regiments and seek ways to improve the efficacy and safety of PTCL, we retrospectively compared the outcomes of 52 PTCL patients treated with CEAC (lomustine, etoposide, cytarabine and cyclophosphamide; n = 28), BEAM (carmustine, etoposide, cytarabine and melphalan; n = 14) and IEAC (idarubicin, etoposide, cytarabine and cyclophosphamide; n = 10) regimens followed by ASCT at our center between 2012 and 2021. Although the time of neutrophil engraftment in CEAC group was earlier than that in IEAC group (*P* = 0.042) and platelet infusion in BEAM group was significantly more than CEAC group (*P* = 0.042), there were no significant difference in platelet engraftment, hematopoietic engraftment and red blood cells infusion among the 3 groups. The transplantation related mortality rate (TRM) and the early overall response rate (ORR) was 3.8% and 85.7% respectively. The 5-year OS and PFS was 62.8% (95% CI: 54.8–70.8%) and 61.0% (95% CI: 53.1–68.9%) respectively. There was no significant difference in TRM, ORR and survival among the 3 groups. Univariate and multivariate analysis showed that high PIT score (the T cell lymphoma prognostic index, > 1) and failure to reach complete response (non-CR) at 3 months after ASCT were common risk factors for OS (*P* = 0.036 and 0.007) and PFS (*P* = 0.021 and 0.012). In conclusion, CEAC and IEAC regimen can be used as alternative conditioning regiments for ASCT in PTCL patients, and their efficacy and safety are comparable to BEAM regiment. Patients with high PIT score and non-CR early after ASCT had worse outcomes.

## Introduction

Peripheral T-cell lymphoma (PTCL) is a group of highly aggressive and heterogeneous non-Hodgkin's lymphoma (NHL), accounting for 10–15% of NHL^1,2^. At present, its first-line treatment regimens are mostly CHOP like regimens. In addition to the good prognosis of anaplastic lymphoma kinase positive anaplastic large cell lymphoma (ALK + ALCL), the other PTCL subtypes have poor response to chemotherapy and are prone to relapse, and the long-term survival rate is less than 30%, which is significantly worse than B-NHL^2–4^. Available retrospective and prospective data suggest that high-dose chemotherapy combined with autologous stem cell transplantation (ASCT) can improve survival in patients with chemotherapy-sensitive diseases, both during front and salvage therapy^5–8^. High-dose chemotherapy (HDC) regimen is an important step in ASCT, which aims to further kill tumor cells in patients and improve the depth of remission, but it also inevitably brings some toxic and side effects. Conditioning regimens for ASCT in PTCL patients were consistent with those commonly used in NHL, including BEAM (carmustine, etoposide, cytarabine and melphalan), BEAC (carmustine, etoposide, cytarabine and cyclophosphamide), CBV (cyclophosphamide, carmustine and etoposide) and so on^7,9,10^. Due to the shortage of carmustine and melphalan in China, CEAC (lomustine, etoposide, cytarabine and cyclophosphamide) has become a widely used conditioning regimen for lymphoma^11^. In addition, our center also innovatively used IEAC (idarubicin, etoposide, cytarabine and cyclophosphamide) as a conditioning regimen for ASCT in lymphoma patients, and previous data have verified its efficacy and safety^12^. However, for PTCL (except ALK + ALCL), no comparison of the efficacy and safety of CEAC, BEAM or IEAC has been reported. This study retrospectively analyzed the efficacy, safety and prognostic factors of ASCT in 52 patients with PTCL who received the above three conditioning regiments, aiming to explore appropriate conditioning regiments and seek ways to improve the efficacy of PTCL.

## Patients and methods

### Patients

**Figure 1:**
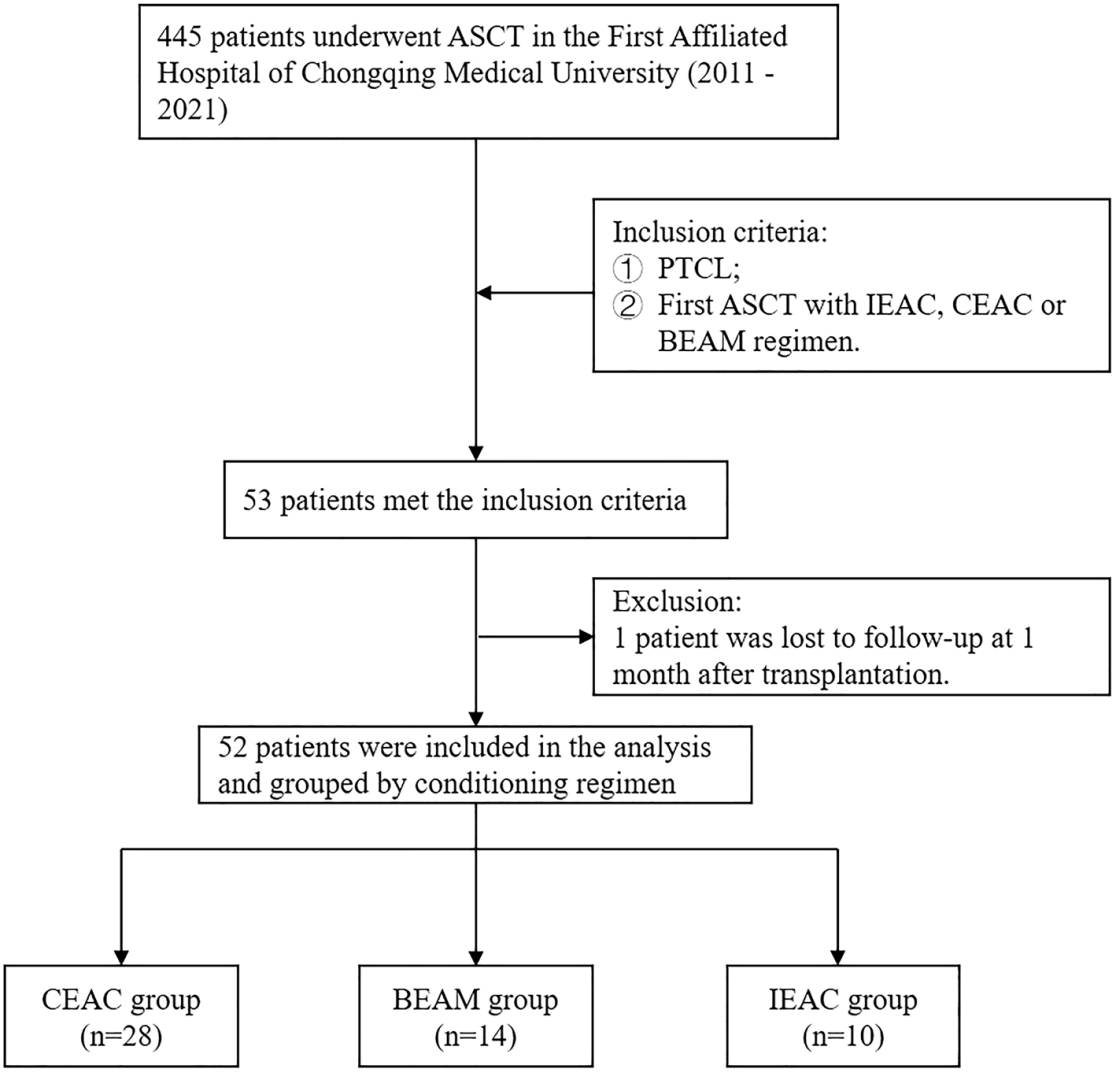
Diagram showing the flow of study participants.

Patients diagnosed with PTCL according to WHO 2016 lymphoma diagnostic criteria^13^ and treated with CEAC, BEAM or IEAC regimen followed by the first ASCT between 2011 and 2021 were eligible for this study. If patients had other tumors prior to ASCT or was lost to follow-up within 3 months after ASCT, they will be excluded. A total of 52 patients were included in the analysis according to the above criteria. According to the conditioning regimen, patients were divided into CEAC group (n = 28), BEAM group (n = 14) and IEAC group (n = 10) (Fig. 1).

### Treatments

#### Before transplantation

Natural killer (NK)/T cell lymphoma (NK/TCL) was dominated by SMILE, GEMOX and CHOP like regimens. For the other subtypes, hyper-CVAD and CHOP like regimens were adopted. Some were combined with high-dose methotrexate, pegaspargase/l-asparaginase, chidamide and radiotherapy.

In addition to 2 patients receiving direct mobilization of recombinant human granulocyte colony stimulating factor (rhG-CSF) 5–10 µg/kg/d, all the other patients received chemotherapy combined with rhG-CSF mobilization. The total number of CD34^+^ cells collected was required to be more than 2 × 10^6^/kg. Finally, the collected peripheral blood hematopoietic stem cells were frozen at − 80 °C.

#### Conditioning regimens

CEAC regimen: lomustine (200 mg/m^2^ on day − 6), etoposide (100 mg/m^2^ q12h or qd on days − 5 to − 2), cytarabine (200 mg/m^2^ q12h or qd on days − 5 to − 2) and cyclophosphamide (1.5 g/m^2^ on days − 5 to − 2). BEAM regimen: carmustine (300 mg/m^2^ on day − 6), melphalan (140 mg/m^2^ on day − 2), the usage and dosage of etoposide and cytarabine are the same as those in CEAC regimen. IEAC regimen: idarubicin (12 mg/m^2^ on day − 6), other drugs are the same as CEAC regimen.

#### Other treatments

Patients received routine bacterial and fungal prophylaxis, antiemetic, hydration and alkalization of urine. Low molecular weight heparin and alprostadil were routinely used to prevent veno-occlusive disease. If patients received high doses of cyclophosphamide, intravenous injection of mesna was required to prevent hemorrhagic cystitis. RhG-CSF was administered starting at day + 1 after transplantation and continued until white blood cells ≥ 4.0 × 10^9^/L. Recombinant human thrombopoietin or recombinant human interleukin-11 was administered starting at day + 4 after transplantation and continued until platelet (PLT) ≥ 100 × 10^9^/L or the treatment course reached 14 days. Blood routine was monitored, and PLT and suspensions of red blood cells (RBCs) were given when necessary.

#### Evaluation points and definitions

The Ann Arbor stage criterion was used for PTCL clinical staging, the ECOG score was used for physical status, and the T cell lymphoma prognostic index (PIT) was used for prognostic risk stratification^14^.

Hematopoietic engraftment (HE) related standards refer to literature^15^: Neutrophil engraftment (NE) was defined as the first of 3 consecutive days with neutrophil count (NEC) > 0.5 × 10^9^/L without rhG-CSF treatment, PLT engraftment (PE) was defined as the first of 3 consecutive days with PTL > 20 × 10^9^/L without transfusion support, and HE was defined as both NE and PE.

According to Lugano 2014 criteria, the therapeutic evaluation was divided into complete response (CR), partial response (PR), stable disease (SD) and progressive disease (PD)^16^. The early response of transplantation was evaluated using the same criteria at 3 months after stem cell transfusion. The overall response rate (ORR) was defined as the sum of the percentage of response to CR and PR. Treatment-related adverse events (TRAEs) were graded according to the National Cancer Institute Common Terminology Criteria for Adverse Events (version 5.0).

Overall survival (OS) was estimated from the time of ASCT until death or the last follow-up. Progress free survival (PFS) was estimated from the time of ASCT until PD, death or the last follow-up. Transplantation related mortality (TRM) was defined as any death occurring without PD within 100 days after transplantation. Lymphoma-related mortality (LRM) was defined as death due to PD or relapse of lymphoma. Non-relapse mortality (NRM) was defined as death from other cause than T-NHL PD or relapse.

### Statistical analysis

All data was statistically analyzed using SPSS (IBM Corp. Released 2017. IBM SPSS Statistics for Windows, Version 25.0. Armonk, NY: IBM Corp). Comparisons in different groups were tested by the Chi-square test, Fisher’s exact tests or Kendall’s W test. OS and PFS were analyzed using the Kaplan–Meier curve and differences among the three groups were assessed by Tarone-Ware test. The Cox proportional hazards model was used for univariate and multivariate analysis. A *P* value < 0.05 was considered significant.

### Ethics approval and consent to participate

This study was approved by the Clinical Research Ethics Committee of the First Affiliated Hospital of Chongqing Medical University and all methods were performed in accordance with the relevant guidelines and regulations.

## Results

### Clinical characteristics

Among the 52 patients with PTCL, there were 34 males and 18 females. The median age was 39 years (13–65 years). Primary diseases included NK/TCL (n = 20), PTCL (not otherwise specified, PTCL-NOS) (n = 10), ALK negative anaplastic cell lymphoma (ALK-ALCL) (n = 10), angioimmunoblastic T-cell lymphoma (AITL) (n = 9) and cutaneous T-cell lymphoma (CTCL) (n = 3). Five patients were hepatitis B carriers with normal liver function, 2 patients had type 2 diabetes mellitus and 3 patients had hyperlipidemia. They received a median of 4 (2–15) cycles of chemotherapy, and 14 patients received local radiotherapy. Patients’ characteristics for each group are summarized in Table 1. There were no significant differences between the three groups (Table 1).

**Table 1:** Clinical characteristics of 52 patients with PTCL in CEAC, BEAM and IEAC groups.

Clinical characteristics	CEAC group n = 28	BEAM group n = 14	IEAC group n = 10	χ^2^	*P*
**Gender**					
Male	20 (71.4%)	8 (57.1%)	6 (60.0%)	1.134	0.675
Female	8 (28.6%)	6 (42.9%)	4 (40.0%)		
**Age, year**					
≤ 60	27 (96.4%)	12 (85.7%)	10 (100.0%)	2.146	0.264
> 60	1 (3.6%)	2 (14.3%)	0 (0.0%)		
**Diseases**					
AITL	4 (14.3%)	2 (14.3%)	3 (30.0%)	5.990	0.665
ALK-ALCL	5 (17.9%)	4 (28.6%)	1 (10.0%)		
CTCL	2 (7.1%)	1 (7.1%)	0 (0.0%)		
NK/TCL	9 (32.1%)	6 (42.9%)	5 (50.0%)		
PTCL-NOS	8 (28.6%)	1 (7.1%)	1 (10.0%)		
**B-symptom**					
Yes	10 (35.7%)	8 (57.1%)	7 (70.0%)	4.101	0.156
No	18 (64.3%)	6 (42.9%)	3 (30.0%)		
**Ann Arbor stages**					
I–II	7 (25.0%)	2 (14.3%)	1 (10.0%)	1.099	0.714
III–IV	21 (75.0%)	12 (85.7%)	9 (90.0%)		
**LDH at diagnosis**					
≤ 250 U/L	13 (46.4%)	5 (35.7%)	3 (30.0%)	1.000	0.648
> 250 U/L	15 (53.6%)	9 (64.3%)	7 (70.0%)		
**Bone marrow involvement**					
Yes	7 (25.0%)	1 (7.1%)	1 (10.0%)	2.089	0.428
No	21 (75.0%)	13 (92.9%)	9 (90.0%)		
**PIT score**					
0–1	15 (53.6%)	7 (50.0%)	8 (80.0%)	2.573	0.310
> 1	13 (46.4%)	7 (50.0%)	2 (20.0%)		
**Radiotherapy**					
Yes	9 (32.1%)	1 (7.1%)	4 (40.0%)	4.188	0.131
No	19 (67.9%)	13 (92.9%)	6 (60.0%)		
**Treatment lines before ASCT**					
1	23 (82.1%)	9 (64.3%)	8 (80.0%)	1.757	0.450
≥ 2	5 (17.9%)	5 (35.7%)	2 (20.0%)		
**Disease status before ASCT**					
CR	18 (64.3%)	9 (64.3%)	5 (50.0%)	4.207	0.381
PR	8 (28.6%)	3 (21.4%)	2 (20.0%)		
PD	2 (7.1%)	2 (14.3%)	3 (30.0%)		

AITL, angioimmunoblastic T-cell lymphoma; ALK-ALCL, anaplastic lymphoma kinase negative anaplastic cell lymphoma; CTCL, cutaneous T-cell lymphoma; NK/TCL, natural killer/T cell lymphoma; PTCL-NOS, peripheral T-cell lymphoma, not otherwise specified; LDH, lactate dehydrogenase; PIT, the T cell lymphoma prognostic index; ASCT, autologous stem cell transplantation; CR, complete response; PR, partial response; PD, progressive disease.

### Transplantation and hematopoietic engraftment

The median number of CD34 + cells was 6.34 (2.00–25.23) × 10^6^/kg, with no statistical significance among the three groups (*P* = 0.076). Except for one patient who died before HE, HE was achieved in all patients with a median time of 13 (9–43) days after ASCT, and the median time of NE and PE was 11 (8–33) and 13 (8–43) days after ASCT, respectively. Although the time of NE in CEAC group was earlier than that in IEAC group (*P* = 0.042), there was no significant difference in PE or HE time among the three groups. In terms of PLT infusion, BEAM group was significantly more than CEAC group (*P* = 0.042), but there was no significant difference in the amount of red blood cells infusion among the three groups (Table 2).

**Table 2:** Transplant-related results in 51 patients.

Variables median (range)	CEAC group n = 27*	BEAM group n = 14	IEAC group n = 10	*P* value
CD34 + cells, 10^6^/kg	8.06 (2.00–25.23)	4.39 (2.20–17.20)	4.36 (2.00–17.11)	0.076
NE, day	10 (8–16)	11 (9–33)	12 (10–17)	0.034
PE, day	13 (8–31)	15 (10–43)	13.5 (10–18)	0.371
HE, day	13 (9–31)	15 (10–43)	14 (11–18)	0.373
HB before regimen, g/L	107 (77–149)	109 (69–149)	108 (88–138)	0.953
PLT before regimen, 10^9^/L	164 (71–283)	154 (11–310)	197 (83–272)	0.696
Red blood cells injected, U	0 (0–6)	1 (0–19.5)	0 (0–4)	0.434
PLT injected, U	3 (1–12)	4.5 (2–6)	3 (1–5)	0.038

*One patient in CEAC group was excluded from the analysis due to death before HE.

NE, Neutrophil engraftment; PE, platelet engraftment; HE, hematopoietic engraftment; HB, hemoglobin; PLT, platelet.

### TRAEs

The most common non-hematologic TRAEs (grade 3–4) occurred during treatment were infection (44.2%), oral mucositis (19.2%), cardiotoxicity (5.8%) and liver damage (not caused by hepatitis B, 3.8%). There was no statistical difference in the incidence of these TRAEs among the three groups (*P* > 0.05) (Table 3). After active symptomatic treatment, except for 2 TRM cases (died of cardiotoxicity) in CEAC group, the TRAEs of other patients gradually improved or even disappeared. The overall TRM rate was 3.8%, and there was no significant difference among the three groups (*P* = 0.704).

**Table 3:** TRAEs within 100 days after ASCT in CEAC, BEAM and IEAC groups.

Adverse events* grade 3–4	CEAC group n = 28	BEAM group n = 14	IEAC group n = 10	*P* value
Infection	10 (35.7%)	9 (64.3%)	4 (40.0%)	0.252
Oral mucositis	4 (14.3%)	3 (21.4%)	3 (30%)	0.458
Cardiotoxicity^‡^	2 (7.1%)	0 (0.0%)	1 (10.0%)	0.583
Liver damage	2 (7.1%)	0 (0.0%)	0 (0.0%)	0.704

*Hematological adverse events were not included. ‡Two patients in the CEAC group died of cardiotoxicity.

### Early response

A total of 49 patients were followed up for more than 3 months after ASCT. Their early response showed that 30 patients who achieved CR before ASCT still maintained CR at 3 months after transplantation. Among the 12 patients with PR before ASCT, 5 had CR, 4 remained in PR, and the remaining 3 had PD at 3 months after transplantation. Among the 7 patients with PD before ASCT, 3 months after transplantation, 1 had CR, 2 had PR and 4 had persistent PD. The early ORR rate was 85.7% (42/49) and there was no significant difference between the three groups (*P* = 0.590).

### Survival analysis

**Figure 2:**
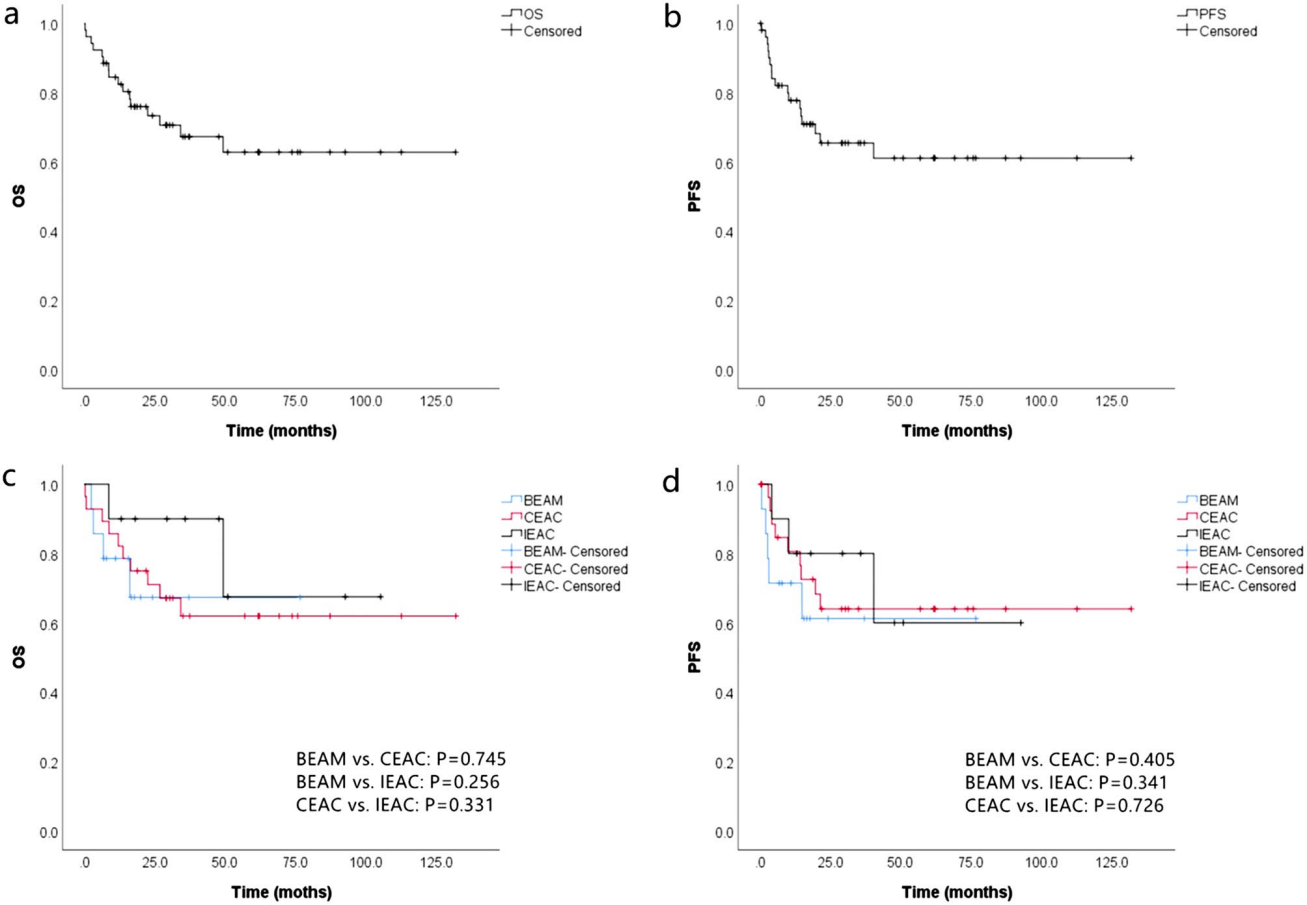
Kaplan–Meier curves of survival. The OS (**a**) and PFS (**b**) curves of 52 patients with PTCL. The OS (**c**) and PFS (**d**) curves of PTCL patients in CEAC (n = 28), BEAM (n = 14) and IEAC (n = 10) groups.

At a median follow-up of 29.1 (0.3–132.1) months, 17 patients (32.7%) had PD and 16 patients died (30.8%), with a TRM of 3.8% (2/52), a LRM of 21.2% (11/52) and a NRM of 1.9% (3/158, died of severe pneumonia) within 100 days after ASCT. The 5-year OS and PFS was 62.8% (95% CI: 54.8–70.8%) and 61.0% (95% CI: 53.1–68.9%) respectively. No significant difference was found among the three groups (Fig. 2).

### Prognosis factors

**Figure 3:**
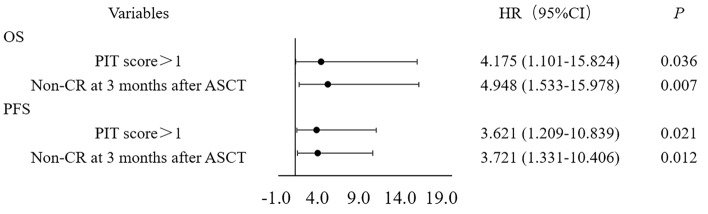
Multivariate analysis of factors potentially associated with OS and PFS.

COX regression analysis was performed on some factors that might affect the prognosis. Univariate analysis showed that PIT score and non-CR at 3 months after ASCT were significantly correlated with OS (*P* = 0.005, 0.014 and 0.001) and PFS (*P* = 0.003 and 0.002), but PD before ASCT was only related to OS (*P* = 0.014) (Table 4). Multivariate analysis showed that PIT score and non-CR at 3 months after ASCT were common risk factors for OS (*P* = 0.036 and 0.007) and PFS (*P* = 0.021 and 0.012) (Fig. 3).

**Table 4:** Univariate analysis of factors potentially associated with OS and PFS.

Variables	OS		PFS	
	HR (95% CI)	*P*	HR (95% CI)	*P*
B-symptom	0.880 (0.327–2.366)	0.800	0.585 (0.216–1.583)	0.291
Ann Arbor stages III/IV	2.166 (0.491–9.566)	0.308	0.920 (0.350–2.418)	0.865
PIT score > 1	5.164 (1.660–16.065)	0.005	4.944 (1.730–14.130)	0.003
PD before auto-HSCT	3.819 (1.311–11.132)	0.014	3.026 (0.975–9.389)	0.055
BEAM regimen	1.402 (0.442–4.452)	0.566	1.595 (0.555–4.586)	0.386
Non-CR at 3 months after ASCT	6.915 (2.238–21.363)	0.001	4.923 (1.825–13.283)	0.002

PIT, the T cell lymphoma prognostic index; PD, progressive disease; CR, complete response; ASCT, autologous stem cell transplantation.

## Discussion

Currently, the treatment of PTCL is still based on combined chemotherapy, and sequential ASCT is an important treatment for PTCL. Although conclusions about the impact of ASCT on prognosis in different centers are different due to differences in disease subtype, stage, chemotherapy regimens and pre-transplant response, etc., most studies support those patients with PTCL could benefit from ASCT^4,8,10,17^. For patients suitable for transplantation, sequential ASCT consolidation therapy is recommended for patients whose response ≥ PR after 4–6 cycles of chemotherapy^18^. In a single-center retrospective study of 58 PTCL patients who underwent ASCT, their 5-year OS and PFS were 53% and 44% respectively, and the 5-year OS was 49% even for elderly patients (≥ 60 years)^19^. Recent studies have shown that the 5-year OS of PTCL patients treatment with ASCT can reach 40–74%, and the 5-year PFS can reach 35–65%^20–22^. In China, due to the lack of nitrosoureas and melphalan, BEAM regimen was unavailable, so CEAC and IEAC regimens were used in our center to replace it. Previously published data indicate that the efficacy and safety of CEAC and IEAC regimens are not inferior to those of BEAM regimen in malignant lymphoma^11,12^, but a detailed comparative analysis of PTCL was not conducted. Fifty-two PTCL patients were included in this study, and the median time of NE and PE after ASCT was 11 (8–33) days and 13 (8–43) days respectively. The time of PE in CEAC group was faster than that in IEAC group (*P* = 0.042), while no significant difference in the time of HE was found among the three groups. The HE of the majority of patients was similar to that reported in literature^7^. The 5-year OS and PFS of the patients were 62.8% and 61.0% respectively, which were not lower than those reported in domestic and foreign literatures^8,19^. In addition, whether patients were treatment with CEAC or IEAC regimen, the survival was similar to that of patients in BEAM group (*P* > 0.05). These results suggested that CEAC and IEAC regimens could be used as the replacement of BEAM regimen with similar efficacy.

The common TRAEs of ASCT in PTCL patients are similar to B-NHL, including infection, mucositis, diarrhea, organ function impairment, etc., and the TRM rate is less than 10%^7,23,24^. Anthracyclines commonly used in NHL chemotherapy have dose-related cardiotoxicity, especially in B-NHL. In the treatment of PTCL, it is used less than B-NHL, resulting in fewer cardiotoxicity before ASCT. High-dose cyclophosphamide may also cause very acute cardiotoxicity in NHL patients previously treated with anthracyclines^25,26^, and one possible explanation based on the autopsy is the toxic endothelial damage followed by extravasation of toxic metabolites resulting in interstitial hemorrhage and edema, which may lead to decreased myocardial compliance and diastolic dysfunction^27,28^. Although idarubicin is a new anthracycline with high efficacy and low cardiotoxicity compared to daunorubicin, cardiotoxicity remains a significant concern in patients treated with CEAC and IEAC regimens. In this study, grade 3–4 TRAEs were similar to those reported in the published literature^29^, and the TRM rate was only 3.8%. Although 2 patients who received CEAC regimen died of cardiotoxicity, the incidence of cardiotoxicity and TRM rate were not significantly different among the three groups (*P* > 0.05). Therefore, the toxicity and side effects of CEAC and IEAC regimens are tolerable, and their safety is no worse than BEAM regimen.

Studies have shown that PTCL subtypes, LDH level, bone marrow involvement, PIT score, disease stage, response before ASCT and other factors can affect the prognosis of patients undergoing ASCT^7,9,30^. A retrospective study of 64 patients with relapsed and refractory PTCL showed that in addition to bone marrow involvement and PIT score, CR after ASCT was also a risk factor for prognosis (*P* = 0.014, 0.022 and 0.019)^7^. Multivariate analysis showed that PIT score and non-CR at 3 months after ASCT were common risk factors for OS (*P* = 0.036 and 0.007) and PFS (*P* = 0.021 and 0.012). Although univariate analysis showed PD before ASCT was correlated with OS (*P* = 0.014), multivariate analysis showed that it was not a risk factor for OS. It may be that ASCT, as a way of consolidation treatment, can benefit the survival of patients with PD before ASCT, while those who still fail to achieve CR after transplantation can predict worse disease response to treatment and worse survival.

The limitation of this study was its small size, which make it is difficult to further analyze of various subtypes. In addition, this was not a prospective randomized study, leading to limitations in the results. The relevant results still need to be verified by prospective and randomized studies with large sample sizes. Registration of related clinical trials is ongoing.

## Conclusion

In conclusion, CEAC and IEAC regimen can be used as alternative conditioning regiments for ASCT in PTCL patients, and their efficacy and safety are comparable to BEAM regiment. Patients with high PIT score and failure to reach CR early after ASCT had worse outcomes.

## Data Availability

The datasets used and/or analyzed during the current study available from the corresponding author on reasonable request.
